# Enhancing Slip, Trip, and Fall Prevention: Real-World Near-Fall Detection with Advanced Machine Learning Technique

**DOI:** 10.3390/s25051468

**Published:** 2025-02-27

**Authors:** Moritz Schneider, Kevin Seeser-Reich, Armin Fiedler, Udo Frese

**Affiliations:** 1 Institute for Occupational Safety and Health of the German Social Accident Insurance (IFA), 53757 Sankt Augustin, Germany; 2 RheinAhrCampus, Koblenz University of Applied Sciences, 53424 Remagen, Germany; 3 German Research Center for Artificial Intelligence (DFKI), 28359 Bremen, Germany

**Keywords:** slip, trip, fall, near fall, machine learning, prevention, workplace safety, neural networks

## Abstract

Slips, trips, and falls (STFs) are a major occupational hazard that contributes significantly to workplace injuries and the associated financial costs. The application of traditional fall detection techniques in the real world is limited because they are usually based on simulated falls. By using kinematic data from real near-fall incidents that occurred in physically demanding work environments, this study overcomes this limitation and improves the ecological validity of fall detection algorithms. This study systematically tests several machine-learning architectures for near-fall detection using the Prev-Fall dataset, which consists of high-resolution inertial measurement unit (IMU) data from 110 workers. Convolutional neural networks (CNNs), residual networks (ResNets), convolutional long short-term memory networks (convLSTMs), and InceptionTime models were trained and evaluated over a range of temporal window lengths using a neural architecture search. High-validation F1 scores were achieved by the best-performing models, particularly CNNs and InceptionTime, indicating their effectiveness in near-fall classification. The need for more contextual variables to increase robustness was highlighted by recurrent false positives found in subsequent tests on previously unobserved occupational data, especially during biomechanically demanding activities such as bending and squatting. Nevertheless, our findings suggest the applicability of machine-learning-based STF prevention systems for workplace safety monitoring and, more generally, applications in fall mitigation. To further improve the accuracy and generalizability of the system, future research should investigate multimodal data integration and improved classification techniques.

## 1. Introduction

Slips, trips, and falls (STF incidents) remain a significant occupational hazard, leading to substantial rates of absence from work and adverse outcomes. In 2023, German Social Accident Insurance (DGUV) reported 171,976 STF incidents, resulting in 7 fatalities and 2291 disability benefit cases [1], underscoring the urgent need for effective prevention strategies. Such incidents are particularly prevalent in sectors such as logistics, transport, and traffic.

Recent advancements highlight the potential of machine learning and sensor technologies for mitigating STF risks. Wang et al. improved near-fall detection among older adults by using body-worn sensors, demonstrating enhanced accuracy and reliability [2]. Similarly, Nikolov et al. emphasized the importance of realistic datasets for fall detection, utilizing low-cost, human-like dummies to generate thermal surveillance data [3].

Innovative fall detection methods continue to emerge. Hsu et al. enhanced detection accuracy in complex environments by utilizing a generative adversarial network (GAN) in a sequence-to-sequence denoising approach, which effectively encodes spatiotemporal correlations for more robust feature extraction [4]. Rahemtulla et al. introduced wearable electronic textiles capable of continuous fall and near-fall detection [5]. Schumann et al. further advanced robustness in fall detection by integrating kinematic data with environmental factors, employing gait analysis and machine learning to identify fall risks in individuals with multiple sclerosis [6].

Despite these advancements, the workplace applicability of current approaches remains limited, owing to their reliance on simulated falls—intentionally induced falls performed in controlled settings, often performed by trained participants or mannequins. This research addresses these gaps by utilizing kinematic data from actual STF events involving physically demanding occupations. Near-falls, characterized by tripping, slipping, or misstepping without falling, are key indicators of future STF risks. Building on the Prev-Fall dataset, this study examines kinematic responses to unexpected gait perturbations in parcel delivery workers and steelworkers [7]. The dataset offers diverse, real-world data, addressing the limitations of earlier studies constrained by simulated falls or narrow demographics.

To enhance detection capabilities, the Prev-Fall dataset was employed in this study to train machine-learning algorithms for near-fall detection. While early-threshold-based classifiers yielded limited success, advanced machine-learning models substantially improved accuracy and reliability. These advancements underscore the necessity of high-quality, realistic data for developing robust STF prevention systems.

### Dataset Differentiation

STF prevention relies on two primary types of datasets: those collected via wearable sensors and those captured with ambient sensors. Ambient sensors, such as camera systems and pressure-sensitive floors, face limitations, such as dependency on camera angles, lighting conditions, and their inability to record precise biomechanical data. In contrast, wearable sensors, particularly inertial measurement units (IMUs), offer continuous monitoring and capture accurate kinematic data, which are crucial for effective fall detection and prevention. This research leverages the Prev-Fall dataset [7] collected through wearable sensors to develop machine-learning models that are both robust and reliable, ensuring suitability for real-world occupational settings.

The primary aim of this work is to perform a neural architecture search with models proven in the field of fall and near-fall detection to analyze their potential on the non-simulated near-fall data of working-age individuals of the previously published Prev-Fall dataset [7]. In order to find appropriate models, we first analyze other works in the field of fall and near-fall detection. We begin by providing an overview of existing fall and near-fall datasets and network architectures used in fall and near-fall detection. Next, we performed linear discriminant analysis and a correlation analysis of the Prev-Fall dataset [7] to gain a deeper understanding of the data. We then perform the neural architecture search before finally testing the best networks on data recorded with the same motion-capture system but in another context and not forming part of the Prev-Fall dataset.

By utilizing kinematic data from real, non-simulated STF incidents, this study seeks more accurate and reliable near-fall detection systems than those common in the field and trained on data from simulated falls [8]. This should ultimately lead to fall detection systems that can improve workplace safety by being able to forecast falls reliably and thereby reduce harm and notify emergency services or by detecting patterns in near-fall events throughout the workday, which can then be combated with traditional fall prevention measures.

To identify the optimal strategy, the study employs a systematic evaluation of multiple neural network architectures. This approach involves fine-tuning network configurations to determine the best-performing models for the dataset, which features data from 17 IMU sensors. Furthermore, the research focuses on optimizing performance scores independently of hardware constraints, inference time, or training resources. This enables an upper bound to be established for near-fall detection accuracy, based on the dataset’s resolution. The findings will also serve as a benchmark for future studies that aim to reduce sensor requirements or computational demands.

## 2. State-of-the-Art STF Data and Detection

### 2.1. Established Fall and Near-Fall Datasets

Fall detection is a well-established research domain supported by datasets such as SisFall [9], UP-Fall [10], and KFall [11]. In addition to these established datasets, however, many studies rely on data collected independently [12,13]. This reliance introduces challenges, including limited participant numbers and the prevalence of simulated falls [7]. Simulated falls, being biomechanically distinct from real falls, reduce the ecological validity and applicability of the findings derived from such datasets [8]. Near-fall detection, while it remains a growing field, faces similar limitations. Previous studies often feature small participant groups and rely on simulated near-falls, limiting the robustness and generalizability of results.

To address these issues, Hellmers et al. utilized data from the SeFallED and CareFall studies, which featured spontaneous, unpredictable near falls induced on a perturbation treadmill. Participants were equipped with six IMU sensors capturing accelerometer and gyroscope data. These studies included a substantial sample size of 87 participants with a median age of 73 years, making them noteworthy contributions to the field [14]. However, the high median age reflects a broader trend in fall research to focus predominantly on older adults, owing to their increased risk of falling. This age bias poses challenges for generalizability. As Sucerquia, López, and Vargas-Bonilla demonstrated, biomechanical differences in the fall data between young and older participants are significant, which can impact classifier performance when this data is applied across age groups [9].

This study relies on the Prev-Fall dataset to address this gap, providing an ideal resource for studying near falls among working-age individuals [7,15]. This dataset includes data from 110 participants, equipped with 17 IMUs that capture accelerometer, gyroscope, and magnetometer data. This level of detail enables a comprehensive analysis of biomechanical processes during both the near-fall and recovery phases, making it a valuable asset for advancing near-fall detection systems.

### 2.2. State-of-the-Art Fall and Near-Fall Detection

Fall and near-fall detection is typically framed as a binary classification problem, where models must identify whether or not an activity constitutes a fall/near fall. In some cases, this is extended to a three-class problem, distinguishing between falls, near falls, and activities of daily living (ADLs) [5]. To be relevant in real-world applications, fall detection must operate in real-time or near real-time, enabling timely interventions such as alerting emergency services [16]. Two primary approaches exist for real-time fall detection. Threshold-based classifiers, which immediately output results when predefined thresholds are exceeded as single points of data arrive, offer simple and fast solutions but lack context. A more robust alternative involves the use of a sliding window to preprocess data, allowing models to generate intermediary predictions as chunks of data stream in [17]. When near-fall detection is combined with fall detection, real-time processing is essential, to provide situational awareness and timely alerts. However, in scenarios such as workplace safety monitoring, near-fall detection can also be performed offline to analyze temporal or spatial patterns.

Threshold-based classifiers, while historically prevalent, have become less common in recent studies, owing to their limitations in handling complex patterns [9]. Modern approaches increasingly rely on machine-learning techniques, such as k-nearest-neighbor (k-NN) classifiers [18,19,20], decision trees [18,19,21], and support vector machines (SVMs) [11,14,19]. These models often require feature engineering, demanding significant domain expertise. By contrast, neural networks excel at learning features directly from raw data, a critical advantage in near-fall detection, where no standardized ergonomic definition exists [22]. Their adaptability makes them ideal for capturing complex biomechanical patterns. Examples of such neural networks are:

- Convolutional neural networks (CNNs): CNNs are effective at detecting local patterns in data. Variants such as the tiny-CNN [23] and DAG-CNN [24] demonstrate impressive speed and accuracy in fall detection tasks;
- Residual neural networks (ResNets): ResNets address the vanishing gradient and degradation problems [25] and have been successfully applied to fall detection [26];
- Hybrid architectures such as the convolutional long short-term memory (convLSTM) combine the advantages of both local pattern recognition and time dependency tracking [14];
- InceptionTime: designed specifically for time-series classification. InceptionTime offers strong potential for fall and near-fall detection with the use of wearable sensors, though it remains underexplored in this domain [2,27].

Although transformer and diffusion-based models have shown encouraging results in natural language processing and computer vision, their applicability to wearable sensor-based fall detection is still largely unknown. Furthermore, their high processing complexity makes them less useful for real-time occupational safety applications. Leveraging the characteristics of the Prev-Fall dataset, which features high-resolution data from 17 IMUs, this study will perform a neural architecture search to evaluate the performance of CNN, ResNet, convLSTM, and InceptionTime architectures. This approach aims to identify optimal models tailored to the dataset’s unique properties, contributing to the advancement of near-fall detection systems.

## 3. Research Question and Hypothesis

The primary research question addressed in this study is: “How effectively can machine learning approaches classify near-fall events”? The research seeks to evaluate the potential of various machine-learning architectures to enhance the detection and prevention of STFs in workplace environments. To answer this question, the study formulates the following key hypotheses.

First, it is hypothesized that machine-learning algorithms can accurately classify near-fall events. Previous research has shown that machine-learning models outperform threshold-based methods in fall detection tasks. Given the complexity and variability of near-fall events, it is expected that machine-learning models, particularly those employing deep-learning techniques, will demonstrate high performance in distinguishing near-falls from baseline activities.

Second, it is hypothesized that real-world data will enhance the generalizability of near-fall detection models. Many existing studies rely on simulated data, which often fails to fully represent the complexity of real-world STF incidents. By using the Prev-Fall dataset, based on data collected in real workplace environments, this study expects the models to exhibit greater robustness and adaptability across different scenarios and populations. Models trained on real-world data are anticipated to outperform those based on simulated data in terms of accuracy and applicability.

In pursuit of these two hypotheses, this study will experiment with different machine-learning architectures, including CNNs, InceptionTime, convLSTM, and ResNets. The aim is to determine their effectiveness in classifying near-fall events and test the best architectures on data foreign to the Prev-Fall dataset, thereby gauging their generalization power. By rigorously testing and comparing these architectures, the study aims to identify the most effective approach to near-fall detection and advance the implementation of machine-learning-based systems for the purpose of improving workplace safety.

## 4. Methods

### 4.1. Selection and Recruitment of Participants

Participants in the Prev-Fall dataset used in this study were selected and recruited as part of the ENTRAPon research project, which is concerned with high-risk sites for STFs. The cohort consisted of 110 workers aged between 19 and 63, 55 of whom were steelworkers and 55 parcel delivery drivers. Participants had to be in good health and able to perform the exercises without assistance; persons with physical or neurological impairments were not allowed to participate. The study design was reviewed and approved by a committee to ensure compliance with ethical research standards. Furthermore, all participants provided informed consent prior to their inclusion in the study. The study on kinematic responses to gait perturbations (already uploaded) provides details on the recruitment procedure and participant requirements [7,15].

### 4.2. Construction of STF Course and Sensors

A specially constructed STF course was used in the experiment for the creation of the Prev-Fall dataset to replicate real-life STFs in a controlled environment. The course consisted of a 15-m-long boardwalk with eighteen integrated STF traps that caused unexpected STFs. These were divided into three categories: slips, trips, and missteps [15]. They were arranged in such a way that participants could not predict their location or type. All participants wore a full-body measurement suit equipped with 17 Xsens Link IMUs (Enschede, The Netherlands), positioned according to the standard Xsens placement protocol to ensure consistent coordinate system alignment across individuals [7,15]. This standardization minimizes inter-subject variability in sensor orientation and ensures biomechanically consistent data acquisition. These sensors collected magnetometer, gyroscope, and accelerometer data at a sampling rate of 120 Hz [7,15]. To the best of our knowledge, there is no comparable real-world dataset that captures near-fall incidents in occupational settings. This dataset was specifically designed to address the limitations of previous studies that rely on simulated falls, thus ensuring greater ecological validity [7,15].

### 4.3. Data Exploration

In order to further deepen the understanding of the Prev-Fall dataset [7], we first used a non-class-specific (S, T, F, and walking) linear discriminant analysis [28,29] to reduce the dimensionality of our data and visualize it. For this approach, we considered the sensor data and the ergonomic metrics separately, creating plots for each.

Furthermore, we performed a detailed correlation analysis, employing cross-correlation to gauge the importance of features for classification: a relevant feature has a high correlation with the target variable but a low correlation with other features [30]. The analysis also shows the potential impact of multicollinearity on the classification of this dataset [31] by calculating the level of the features’ mutual correlation. Lastly, cross-correlation has been used to detect data overlap by showing the level of similarity between the time series from different classes. Similar time-series data belonging to different classes have the potential to complicate the finding of a clean decision boundary [32]. The cross-correlation analysis was conducted to evaluate three primary aspects:

1. Between-feature correlation: first, we grouped the sensor data based on biomechanical characteristics: upper body, lower body, head, and others (e.g., CoM). Second, we calculated a pairwise cross-correlation between all features of one STF trial (e.g., Trial 1: left toe acceleration vs. right wrist velocity), then summed and averaged the cross-correlations across all STF trials. This resulted in a very large correlation matrix. We, therefore, further summed and averaged them across the aforementioned biomechanical characteristics and created a correlation dendrogram;
2. Within-class correlation: we calculated the average multivariate cross-correlation between each pair of STF trials from the same STF class;
3. Between-class correlation: we calculated the average multivariate cross-correlation for each pair of STF trials from different classes.

Specifically, we calculated the zero-normalized cross-correlation in order to detect structural patterns, taking the maximum correlation value for all valid time shifts. The calculation was performed using ObsPy [33].

### 4.4. Neural Architecture Search

In order to find a high-performing network architecture for the Prev-Fall dataset, we systematically tested different configurations of suitable network types for different window sizes. The windows contain 493 channels made up of Xsens data, including acceleration, velocity, angular acceleration and angular velocity, body angles, sensor and CoM positions, and foot contact information. We ran experiments with 500 ms, 1000 ms, and 1500 ms windows with a step size of 50% and tagged a window as belonging to an STF event if it contained 30 data points of the STF event. For the sampling rate of 120 Hz, this is equivalent to 250 ms of the STF event. To balance the significantly larger number of windows containing baseline walking on the STF course with the windows containing STF events, we rebalanced the dataset before training, and windows were randomly drawn until all classes were the size of the smallest class. We subsequently split the data randomly into 70% training and 30% validation datasets.

For the neural architecture search, we implemented an updated version of the mcfly automated deep learning on the time series framework [34]. We trained 50 different CNNs, ResNets, convLSTM, and InceptionTime neural networks for up to 20 epochs. We used early stopping when no improvement in the validation loss was observed for 5 successive epochs. We adjusted the different parameters equivalent to [34] according to the network type.

For all four network types, we utilized an adam optimizer [35] and used random search to find suitable values for weight decay (between 0.1 and 0.0001) and learning rate (between 0.1 and 0.0001). For CNNs, we varied the number of convolutional layers, the number of filters in the convolutional layers, and the number of nodes in the following fully connected layer. For the ResNets, we varied the number of convolutional blocks, the number of filters, and their size. For InceptionTime, we varied the number of inception blocks, the number of filters, and their size. For deepConvLSTMs, we varied the number of initial convolutional layers, the convolutional filter size, the number of lstm layers, and the number of features in the hidden state. The ranges of all network-specific parameters can be found in Appendix A, Table A1. 

To measure network performance, we calculated the accuracy and the weighted F1 score [36] for the training and validation data.

### 4.5. Testing Networks on Foreign Data

Following the neural architecture search, we tested the top three networks of each network type and from all three window sizes on 25 h of as-yet-unseen data from a different dataset recorded with the same Xsens Link system and with 17 IMUs. These data were recorded in the IFA’s ergonomics laboratory and depict a range of ADLs, from walking and filling shelves at different heights with packages of different sizes and weights to using a cordless screwdriver at different heights [37]. The data are not labeled with ADL events, nor do they contain labeled STF events. The activities performed in these recordings are distinctly different from the activities in our STF training and validation set, in addition to the normal activity of walking around. We ran this test to gauge the generalization power of the trained networks with respect to the data from as-yet-unseen environments and to gauge the false-positive rate for as-yet-unseen activities. We, therefore, calculated the false-positive rate as a quantitative performance metric and checked the timestamps of the windows in which the false positives occurred to gain a qualitative impression of which activities lead to false positives.

## 5. Results

### 5.1. Data Exploration

**Figure 1:**
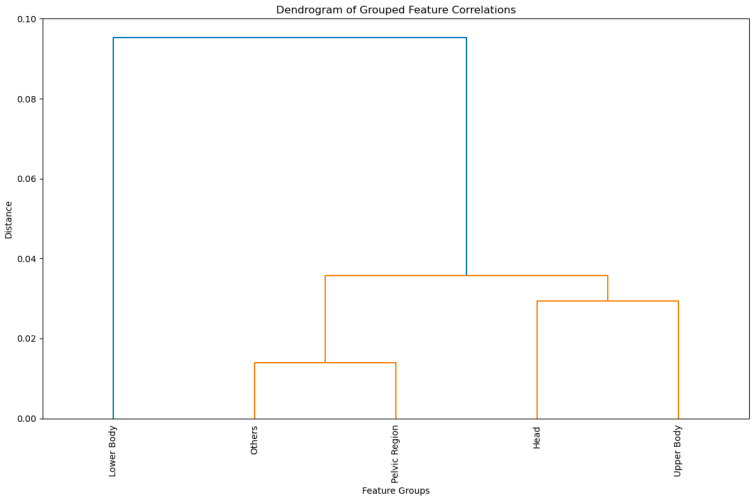
Dendrogram showing the correlation distance between features in different anatomical regions averaged across all trials. The “Others” category contains features that cannot be clearly assigned to specific anatomical regions or sensor modalities. These include whole-body metrics (e.g., center of mass (CoM) measures). The inclusion of “Others” ensures that the analysis captures all potentially relevant feature interrelationships.

To evaluate the quality and redundancy of the sensor data, we analyzed the between-feature correlation. The analysis particularly revealed high correlations between features within the same anatomical region (e.g., left foot and left toe; between the different vertebrae) and for related metrics in the same anatomical region (e.g., left foot acceleration and left foot velocity, joint angles of vertebrae). The correlation between features from different anatomical regions ranged from −0.65 to 0.99 but was on average low, at −0.06 for negative and 0.16 for positive correlations. A Dendrogram of averaged correlations between the different feature groups can be seen in Figure 1.

For the purpose of the neural architecture search, we decided to retain the few features with high intra-group correlations, as they provide complementary information on anatomy and are, thus, critical for model performance and interpretability. Redundancy was intentionally preserved to ensure a comprehensive representation of biomechanical processes, leveraging the robustness of machine-learning models such as InceptionTime and ResNet against correlated inputs.

The results of the intra-class and inter-class correlateon analysis can be seen in Table 1 and Table 2, respectively. They show that the multivariate zero-normalized cross-correlations within a class are only marginally higher than between classes and could suggest that linear relationships are not sufficient for a proper understanding of the complexity of the data.

**Figure 2:**
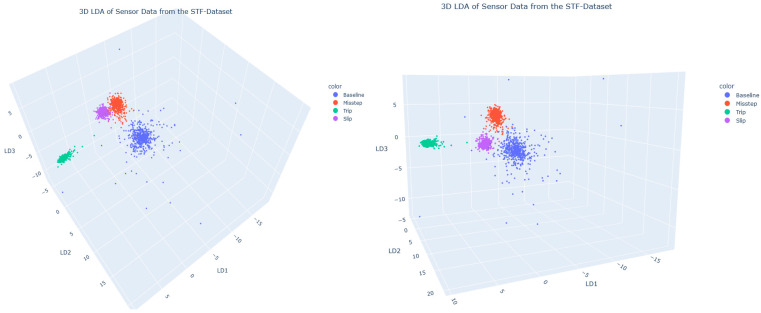
LDA of sensor data of the STF dataset. The two graphs are different perspectives of the same plot.

The two graphs in Figure 2 present the results of a linear Discriminant Analysis (LDA) applied to the Prev-Fall dataset, reducing dimensions and thus yielding distinct clusters for the different event types-namely baseline, misstep, trip, and slip. The primary objective of these visualizations is to assess the extent to which LDA effectively separates these classes in a reduced three-dimensional space, where each axis (LD1, LD2, and LD3) represents one of the discriminant components. The LDA components are not used as the input in further data analysis steps or the neural architecture search. Rather, LDA was employed to reduce the high-dimensional feature space to a lower-dimensional representation, enabling intuitive visualization and facilitating the interpretability of class separability, with the objective of identifying the underlying patterns within the data. The two graphs in Figure 2 show the same LDA from different perspectives. A relatively high separation between the classes is observed, particularly for the trip class (green), which is clearly isolated from the other classes. The Slip (purple) and Misstep (red) classes appear to be more densely packed than the Baseline (blue) class, which scatters the most strongly. Compared to the clear separation seen from the trip cluster, the other three clusters are, at any rate, close together and less differentiated.

### 5.2. Neural Architecture Search

For this study, we implemented a neural architecture search framework, which facilitated automated training and hyperparameter optimization to solve our time-series classification problem. This framework enabled the efficient exploration of multiple deep-learning architectures, including CNN, ResNet, convLSTM, and InceptionTime, with the goal of identifying the most accurate models for detecting near-fall events. Our focus lay on using Xsens sensor data, and we excluded the advanced ergonomic metrics present in the Prev-Fall dataset.

The three best-performing models independent of window size can be seen in Table 3.

Table A2, Table A3 and Table A4 in Appendix B summarize the top-performing models across different model types, ranked by their highest validation F1 score. For each model type, the three models with the best validation performances are reported. Additionally, the tables provide the initial learning rate, which influences the convergence speed, and the regularization rate, which helps to reduce overfitting and improve generalizability.

Each model’s architecture type is specified, distinguishing models according to structural characteristics. Key performance metrics, such as the best F1 score and accuracy achieved in both training and validation, reveal the models’ effectiveness and generalization capability.

CNNs and InceptionTime demonstrated exceptional consistency over all of the window lengths, with the models achieving higher F1 scores correlating with better generalization capabilities and lower validation losses. The model achieving the highest performance in terms of weighted F1 score on the validation dataset is the InceptionTime model, which attained a validation F1 score of 0.9632 at a window size of 500 ms, potentially attributed to its architectural design and hyperparameter tuning, including a learning rate of 0.000197 and a regularization rate of 0.013995.

Some models exhibited slight discrepancies between training and validation metrics. Overall, however, these architectures showcased robustness and reliability in feature extraction and classification tasks.

**Figure 3:**
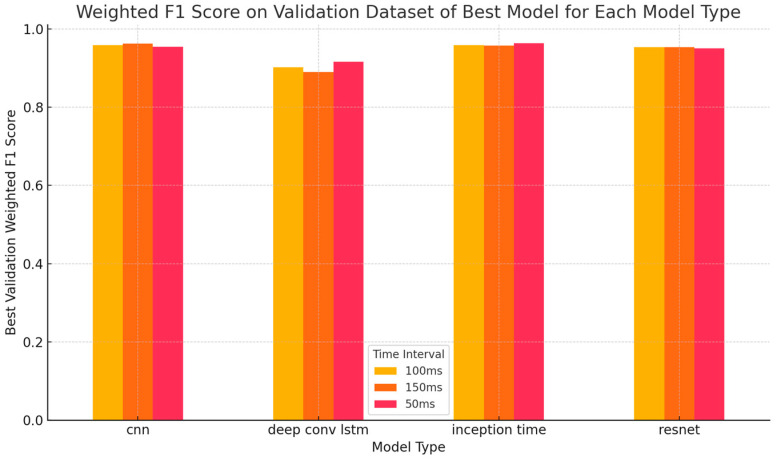
Weighted F1 score on the validation dataset of the best-performing model of each model type for the three different window lengths.

**Figure A1:**
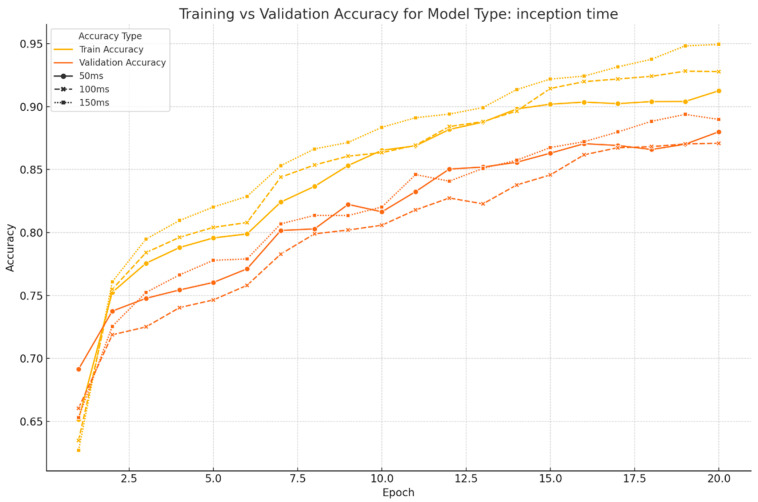
Validation and training accuracy progression during the training of the best-performing InceptionTime model for all three window lengths.

**Figure A2:**
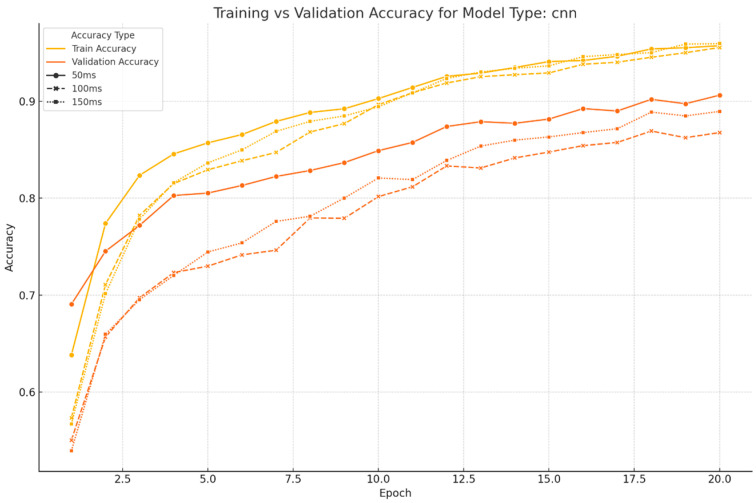
Validation and training accuracy progression during the training of the best-performing CNN model for all three window lengths.

**Figure A3:**
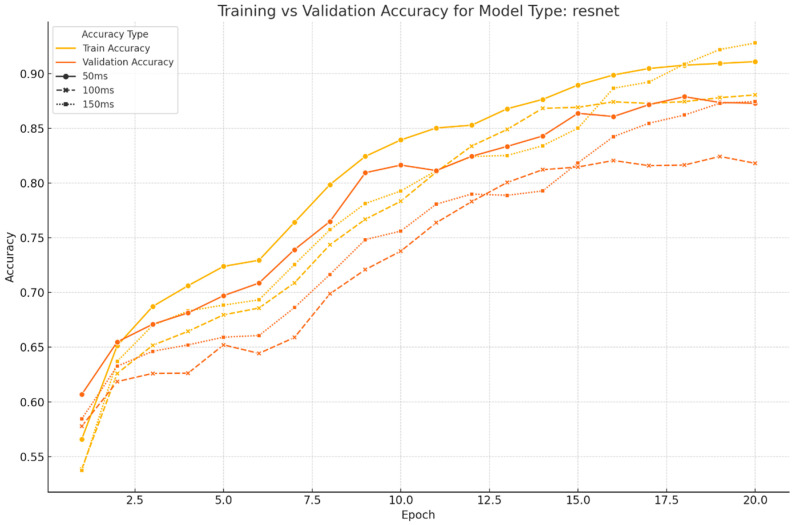
Validation and training accuracy progression during the training of the best-performing ResNet model for all three window lengths.

**Figure A4:**
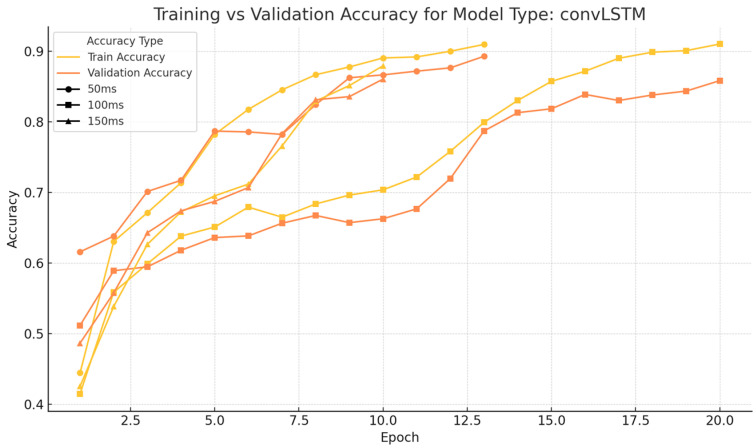
Validation and training accuracy progression during the training of the best-performing convLSTM model for all three window lengths.

Figure 3 shows that the different window sizes impacted the weighted f1 score on the validation dataset only slightly. Figure A1, Figure A2, Figure A3 and Figure A4 in Appendix C further support this observation. They illustrate the progression of training and validation accuracy over epochs for various neural network architectures. The figures in Appendix C are organized by model types, such as CNN and InceptionTime, and further differentiated by time intervals of 500 ms, 1000 ms, and 1500 ms.

**Figure 4:**
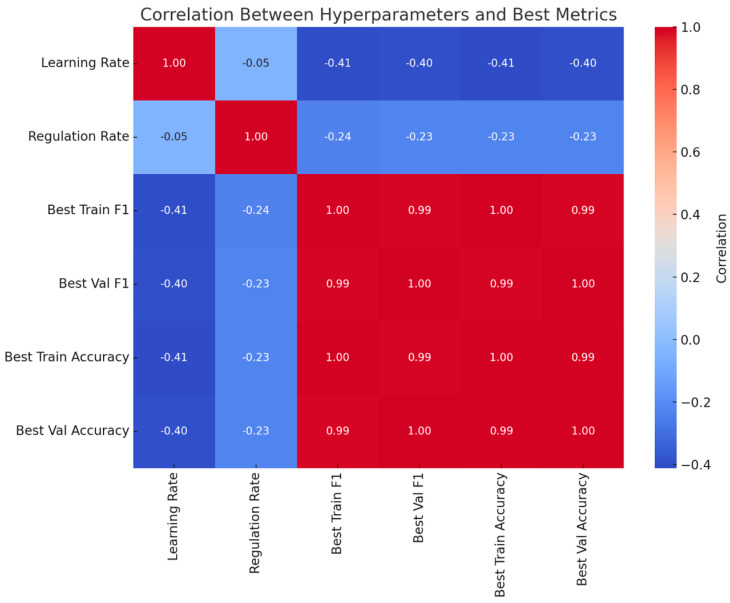
Correlation between hyperparameters and best metrics for ML models.

Figure 4 shows the correlation of the learning and regularization rates with the evaluation metrics of the neural architecture search. All evaluation metrics are highly correlated with each other. More importantly, the learning rate correlates negatively with the evaluation metrics, and the regularization rate correlates slightly negatively with the evaluation metrics. The correlation between the learning and regularization rates is close to zero and spurious, since both are randomized independently.

### 5.3. Test on Foreign Data

The results of testing the networks from the neural architecture search on the Prev-Fall dataset on foreign data can be seen in Table 4, Table 5 and Table 6. The false-positive rate of the networks ranged from 10.1 to 27.8 percent, with performances being considerably better for 500 ms than 1000 ms and 1500 ms windows. Manual investigation revealed that false positives occurred particularly during movements such as bending forward (e.g., lifting boxes), squatting or kneeling (e.g., setting objects down), and raising the hands—especially when the arms were extended outward.

## 6. Discussion

### 6.1. Insights from the Data Analysis

The correlation analysis provided important insights into the interdependence of features across different sensor groups, revealing both redundancy and complementary relationships. As expected, we found especially high correlations for the same metrics in the same region (i.e., velocity of left foot and left toe) and for related metrics such as velocity and acceleration. However, when different metrics from different regions were compared, the averages were generally low (−0.06 for negative and 0.16 for positive correlations).

The low correlation values across most feature groups highlight the independence of these features, underscoring their potential complementary value in classification. For instance, the moderate correlations with only the pelvic region of external features (“Others”) suggest that, while these features may not independently predict STF events, their inclusion probably enhances the robustness of the models by capturing additional variance in the data. The observed low correlations indicate that models such as InceptionTime and CNNs, which excel at feature extraction from raw and heterogeneous inputs, are well-suited for this dataset. Conversely, models requiring manual feature engineering (e.g., SVMs or decision trees) might struggle to leverage this complexity, as they rely on pre-selected, highly correlated features. Future work could investigate whether dimensionality reduction techniques such as PCA or feature selection based on correlation thresholds improve computational efficiency without sacrificing model performance.

The linear discriminant analysis (LDA) demonstrated varying degrees of class separability, with the trip class showing consistent isolation across projections (Figure 2). This indicates that the kinematic signatures of trips are distinct, probably owing to unique biomechanical features such as abrupt forward shifts in the CoM. In contrast, misstep and slip events exhibited significant overlap, reflecting shared instability characteristics such as lateral CoM shifts or irregular gait recovery patterns. The baseline class, which consistently overlaps with both missteps and slips, further complicates classification. This overlap can be attributed to dynamic movements during normal walking, such as rapid directional changes or decelerations, which mimic early indicators of instability. While LDA captured meaningful separability, the overlaps between missteps, slips, and baseline activities highlight the need for additional features or nonlinear techniques. The observed overlaps between missteps, slips, and baseline activities indicate that a simple linear separation of classes is not feasible. Nonlinear approaches, such as neural networks, are therefore required to capture the complex, nonlinear relationships present in the data. The correlation analysis suggests that including biomechanical variables such as joint torques, stride asymmetry, or temporal patterns in CoM trajectory could enhance class separability. Additionally, nonlinear dimensionality reduction methods such as t-SNE [38] could complement LDA by better capturing the nonlinear relationships between features.

### 6.2. Neural Network Performances

Overall, we found that the network types we identified as popular for fall and near-fall detection work well on the Prev-Fall dataset, with no architecture being completely unable to tackle the problem. While the performance of the neural networks on our dataset is lower than on datasets discussed in Section 2, this can be explained by the higher difficulty of detecting near-falls—a more subtle event than falls—and by the differentiation between multiple types of near falls rather than the issue being treated as a binary detection problem.

The neural architecture search highlighted distinct strengths and weaknesses across model types and time windows (Figure 3, Table A2, Table A3 and Table A4). CNNs performed exceptionally well for short time intervals (500 ms), achieving validation F1 scores of up to 0.954. Their ability to capture localized patterns, such as sudden spikes in acceleration or angular velocity, makes them ideal for real-time applications. Surprisingly, their performance barely declined for longer windows (1000 ms, 1500 ms), suggesting that the CNNs’ difficulties in integrating temporal dependencies over extended periods did not meaningfully come into effect here. InceptionTime consistently outperformed other models for longer intervals (1000 ms and 1500 ms), with F1 scores approaching 0.96, but it also performed the best overall for a window size of only 500 ms. Its multiscale temporal feature extraction capabilities probably enabled it to capture both short-term and long-term patterns that are critical for distinguishing between STF events. The architecture’s scalability and robust generalization make it a strong candidate for offline analysis or retrospective evaluations of workplace safety. ResNets demonstrated stable performance across all time intervals, but did not match the F1 scores of InceptionTime. While ResNets effectively addressed issues such as vanishing gradients, their limited ability to capture multiscale temporal patterns may explain their slightly lower performance for STF classification. An analysis of false positives revealed that biomechanical movements such as bending and squatting are often misclassified because their sensor patterns resemble actual falls. This suggests that existing models lack kinematic context integration, which is essential for distinguishing controlled movements from near-fall events. Future improvements could incorporate context-aware kinematic features, such as motion dynamics and postural stability, along with task-specific biomechanical constraints to improve classification accuracy and reduce false positives.

The hyperparameter correlation analysis (Figure 4) revealed that medium learning rates (0.001–0.005) and regularization rates (0.001–0.01) were associated with the best performance. Excessively high learning rates led to unstable models, while low regularization occasionally resulted in overfitting. These findings emphasize the importance of careful hyperparameter tuning to balance model stability and generalization.

### 6.3. False Positives and Generalization

Testing the models resulting from the neural architecture search on as-of-yet-unseen data gives us insights into their generalization abilities. Since this test data was not labeled and did not contain near-falls either way, we measured the false-positive rate to gauge how many false alarms the best networks would send. While the models exhibited promising generalization capabilities on unseen foreign data, capable of achieving false-positive rates below 20%, this would still be too high for real-world applications without further techniques to eliminate erroneous alarms. 

False positives were observed, particularly during biomechanically complex movements such as bending, squatting, or raising the hands. These activities involve significant shifts in the CoM or angular momentum, which resemble instability patterns seen in true near falls. For example, bending forward to lift an object causes the CoM to move anteriorly relative to the base of support (BoS), mimicking the destabilization observed during actual falls. Similarly, raising the hands while extending the arms redistributes mass, creating kinematic patterns that can be mistaken for balance recovery attempts. These findings highlight a critical challenge for real-world deployment: distinguishing between stability-critical movements and true near falls. Incorporating additional contextual data, such as task-specific information or environmental cues, as well as additional ADL data, could help reduce false positives. Alternatively, hybrid approaches that combine machine learning with rule-based systems as common in the fields of computer vision and natural language processing may improve classification reliability by integrating domain knowledge into the decision-making process.

## 7. Conclusions

The findings of this study demonstrate the significant potential of advanced machine-learning models for near-fall detection, particularly in real-world occupational settings. By leveraging high-quality kinematic data from actual STF events, this research bridges critical gaps left by prior studies that relied heavily on simulated datasets or were narrowly focused on older populations. The inclusion of diverse participants, representing physically demanding professions, such as parcel delivery workers and steelworkers, underscores the broader applicability of the proposed methods to working-age individuals—a demographic often under-represented in STF research.

One of the primary practical contributions of this study lies in its evaluation of neural network architectures tailored to time-series data. The consistent performance of CNNs for shorter time intervals highlights their suitability for real-time near-fall detection systems, which are critical in workplace environments where timely intervention can prevent injuries. CNNs’ ability to operate with lightweight computational requirements further strengthens their feasibility for integration into wearable devices, such as smart clothing or footwear with embedded sensors, enabling continuous monitoring without imposing a significant hardware burden. In contrast, the superior performance of InceptionTime models across longer time intervals positions them as ideal candidates for offline analysis or retrospective safety evaluations. This capability is especially valuable for post hoc investigations, allowing safety managers to identify temporal or spatial patterns in STF events and design targeted interventions. For instance, near-fall hot spots within a facility or recurrent hazardous behaviors can be identified and mitigated, improving workplace safety protocols over time.

Despite these advances, challenges remain. The overlap between baseline activities and near falls, as revealed by the LDA results, emphasizes the need for more sophisticated feature engineering or multimodal integration. Future work should explore the inclusion of external contextual data, such as environmental information (e.g., surface type or incline) or additional sensor modalities (e.g., visual data from cameras and force sensors embedded in flooring), as well as the integration of additional contextual features, such as task-specific metadata or ergonomic risk factors, to improve model robustness. In addition, a hybrid approach combining feature engineering and deep learning may help mitigate false positives in biomechanically complex activities. These enhancements could provide a richer context for distinguishing between complex stability-critical movements and true near-fall events, thereby reducing false positives.

Scalability also represents a key area of focus. While the Prev-Fall dataset provides a robust foundation, extending the dataset to include a broader range of occupations, environmental conditions, and demographic profiles would enhance the generalizability of the models. Such expansions would not only improve the reliability of near-fall detection systems across industries but also pave the way for developing population-specific models tailored to different worker groups or workplace risks.

The practical implications of this work extend beyond workplace safety. The methodologies and insights presented here have the potential to inform near-fall detection systems in other domains, such as elder care or rehabilitation. For instance, applying these models in home settings could provide continuous monitoring for at-risk individuals, offering early interventions and personalized fall prevention strategies.

In summary, this study represents a significant step forward in the development of near-fall detection systems, addressing critical gaps in prior research and setting new benchmarks for model performance in real-world settings, and represents one of the first systematic evaluations of deep-learning models for near-fall detection using a real-world dataset, demonstrating the limitations of existing approaches in handling biomechanically complex movements. Our findings provide critical insights for developing robust and context-aware fall prevention systems. The integration of cutting-edge machine-learning architectures with high-quality kinematic data ensures both accuracy and practical applicability. As these systems continue to evolve, their deployment in occupational settings has the potential to drastically reduce STF-related injuries, improving worker safety and productivity on a global scale. By laying the foundation for more robust, generalizable, and context-aware detection systems, this research contributes a transformative framework for advancing occupational safety and injury prevention.

## Figures and Tables

**Table 1:** Within-class correlation.

Class	Correlation Coefficient
Misstep	0.055
Slip	0.058
Trip	0.073

**Table 2:** Between-class correlation.

Class Pair	Correlation Coefficient
Misstep–Slip	0.030
Misstep–Trip	0.015
Slip–Trip	0.040

**Table 3:** Showing the top 3 networks according to the validation-weighted F1 score from the neural architecture search on the rebalanced Prev-Fall dataset.

Model	Window Size	Validation Accuracy	Validation F1 Score
InceptionTime	500 ms	0.9385	0.9632
CNN	1500 ms	0.9298	0.9622
InceptionTime	1000 ms	0.9123	0.9590

**Table 4:** This table shows the false positive rate on as-yet-unseen data spliced into windows with a size of 1500 ms for the best three networks of each network type from the neural architecture search performed on the Prev-Fall-Dataset.

Experiment	Model	Rank	FP Rate
1500 ms	CNN	1	0.2284
	CNN	2	0.2785
	CNN	3	0.1579
	InceptionTime	1	0.1595
	InceptionTime	2	0.1596
	InceptionTime	3	0.2028
	ResNet	1	0.1988
	ResNet	2	0.2211
	ResNet	3	0.2232
	deepConvLSTM	1	0.2151
	deepConvLSTM	2	0.2482
	deepConvLSTM	3	0.2631

**Table 5:** This table shows the false positive rate on as-yet-unseen data spliced into windows with a size of 1000 ms for the best three networks of each network type from the neural architecture search performed on the Prev-Fall-Dataset.

Experiment	Model	Rank	FP Rate
1000 ms	CNN	1	0.2230
	CNN	2	0.1948
	CNN	3	0.2499
	InceptionTime	1	0.2069
	InceptionTime	2	0.2101
	InceptionTime	3	0.1565
	ResNet	1	0.2326
	ResNet	2	0.1982
	ResNet	3	0.1804
	deepConvLSTM	1	0.2418
	deepConvLSTM	2	0.2310
	deepConvLSTM	3	0.2595

**Table 6:** This table shows the false positive rate on as-yet-unseen data spliced into windows with a size of 500 ms for the best three networks of each network type from the neural architecture search performed on the Prev-Fall-Dataset.

Experiment	Model	Rank	FP Rate
500 ms	CNN	1	0.2019
	CNN	2	0.1588
	CNN	3	0.2028
	InceptionTime	1	0.1013
	InceptionTime	2	0.1675
	InceptionTime	3	0.2000
	ResNet	1	0.1813
	ResNet	2	0.1862
	ResNet	3	0.2121
	deepConvLSTM	1	0.2048
	deepConvLSTM	2	0.2257
	deepConvLSTM	3	0.2549

## Data Availability

The data used in this study are available on request from the corresponding author. The data are currently not publicly available, as they still need to be technically prepared for dissemination and, for legal reasons, all participating institutions must be involved in any dissemination.

## Appendix A

**Table A1:** Table containing the hyperparameters randomized for the different model architectures during the neural architecture search.

Model	Hyperparameter	Range
CNN	Number of Layers	1–10
CNN	Number of Filters	10–100
CNN	Number of Fully Connected Nodes	10–2000
ResNet	Number of Residual Blocks	2–5
ResNet	Number of Filters	32–128
ResNet	Kernel Size	8–32
deepConvLSTM	Number of Conv Layers	1–10
deepConvLSTM	Number of Conv Filters	10–100
deepConvLSTM	Number of LSTM Layers	1–5
deepConvLSTM	Size of LSTM Hidden State	10–100
InceptionTime	Number of Inception Modules	3–6
InceptionTime	Number of Filters	32–96
InceptionTime	Kernel Size	10–100

## Appendix B

**Table A2:** Results of neural architecture search with window length of 500 ms.

A.	Model	Learning Rate	Regulation Rate	Best Train F1	Best Val F1	Best Train Accuracy	Best Val. Accuracy	Best Train Loss	Best Val. Loss
500 ms	CNN	0.0050	0.0015	0.9496	0.9542	0.9434	0.9297	0.1621	0.1660
	CNN	0.0005	0.0010	0.9811	0.9536	0.9748	0.9291	0.0968	0.1871
	CNN	0.0006	0.0040	0.9774	0.9519	0.9711	0.9274	0.1061	0.1919
	inception time	0.0001	0.0139	0.9640	0.9632	0.9578	0.9385	0.7960	0.7998
	inception time	0.0009	0.0001	0.9647	0.9573	0.9585	0.9326	0.7791	0.7904
	inception time	0.0011	0.0002	0.9606	0.9550	0.9544	0.9303	0.7853	0.7925
	ResNet	0.0007	0.0001	0.9644	0.9501	0.958	0.9256	0.7815	0.7954
	ResNet	0.0008	0.0008	0.9400	0.9415	0.9339	0.9174	0.8057	0.8048
	ResNet	0.0015	0.0032	0.9422	0.9415	0.9361	0.9174	0.8157	0.8124
	deepConvLSTM	0.0001	0.0022	0.9195	0.9166	0.9134	0.8928	0.2718	0.3176
	deepConvLSTM	0.0002	0.0013	0.9129	0.9119	0.9072	0.8881	0.3059	0.2910
	deepConvLSTM	0.0004	0.0002	0.9028	0.9114	0.8968	0.8875	0.2940	0.3012

**Table A3:** Results of neural architecture search with window length of 1000 ms.

	Model	Learning Rate	Regulation Rate	Best Train F1	Best Val F1	Best Train Accuracy	Best Val. Accuracy	Best Train Loss	Best Val. Loss
1000 ms	CNN	0.0030	0.0023	0.9589	0.9588	0.9487	0.9123	0.1492	0.1588
	CNN	0.0007	0.0010	0.9814	0.9581	0.9710	0.9115	0.099	0.1720
	CNN	0.0004	0.0110	0.9653	0.9523	0.9551	0.9061	0.1841	0.2152
	inception time	0.0025	0.0003	0.9654	0.9590	0.9551	0.9123	0.7810	0.7904
	inception time	0.0016	0.0001	0.9661	0.9539	0.9559	0.9076	0.7792	0.789
	inception time	0.0002	0.0134	0.9549	0.9539	0.9448	0.9076	0.8083	0.8189
	ResNet	0.0006	0.0022	0.9565	0.9531	0.9463	0.9068	0.7982	0.8038
	ResNet	0.0009	0.0002	0.9648	0.9523	0.9545	0.9061	0.7821	0.7950
	ResNet	0.0001	0.0010	0.9749	0.9514	0.9645	0.9053	0.7747	0.8011
	deepConvLSTM	0.0078	0.0001	0.9203	0.9023	0.9101	0.8583	0.2884	0.3421
	deepConvLSTM	0.0002	0.0002	0.8356	0.8246	0.8272	0.7832	0.4865	0.5048
	deepConvLSTM	0.0003	0.0009	0.7923	0.7706	0.7845	0.7339	0.6243	0.6619

**Table A4:** Results of neural architecture search with window length of 1500 ms.

	Model	Learning Rate	Regulation Rate	Best Train F1	Best Val F1	Best Train Accuracy	Best Val. Accuracy	Best Train Loss	Best Val. Loss
1500 ms	CNN	0.0007	0.0002	0.9966	0.9622	0.9917	0.9298	0.0672	0.1875
	CNN	0.0010	0.0006	0.986	0.9559	0.9815	0.9236	0.0721	0.183
	CNN	0.0005	0.0013	0.9874	0.9549	0.9826	0.9227	0.1140	0.1872
	inception time	0.0014	0.0017	0.9710	0.9577	0.9662	0.9254	0.7866	0.7940
	inception time	0.0014	0.0016	0.966767	0.954928	0.962017	0.922735	0.790944	0.802259
	inception time	0.0012	0.0001	0.9743	0.9540	0.9695	0.9218	0.7725	0.7919
	ResNet	0.0001	0.0056	0.9745	0.9530	0.9697	0.9209	0.7772	0.8029
	ResNet	0.0040	0.0001	0.9712	0.9521	0.9664	0.9200	0.7756	0.7958
	ResNet	0.0001	0.0006	0.9736	0.9503	0.9689	0.9182	0.7728	0.7973
	deepConvLSTM	0.0045	0.0002	0.8835	0.8901	0.8793	0.8605	0.3548	0.3344
	deepConvLSTM	0.0002	0.0002	0.8357	0.7503	0.8309	0.7202	0.4545	0.7359
	deepConvLSTM	0.0026	0.0001	0.7098	0.7322	0.7110	0.7131	0.6011	0.5363
